# Correlates of conflict resolution across cultures

**DOI:** 10.1017/ehs.2021.41

**Published:** 2021-08-31

**Authors:** Zachary H. Garfield

**Affiliations:** Institute for Advanced Study in Toulouse, Université de Toulouse 1 Capitole, Toulouse, France

**Keywords:** leadership, mediation, group living, conflict, cross-cultural

## Abstract

Conflicts are ubiquitous between individuals as well as between groups. Effective conflict resolution is essential for individual well-being and group functioning and often involves leadership dynamics. The evolutionary human sciences have suggested that conflict resolution is shaped by psychological heuristics, norms and ecology. There are limited empirical data, however, on conflict resolution across cultures. Using a cross-cultural database of 109 leadership dimensions coded from over 1200 text records from the eHRAF ethnographic database, exploratory analyses investigated correlates of conflict resolution. The results revealed greater evidence of conflict resolution among kin groups than political groups and greater evidence of within-group conflict resolution than between-group, which did not vary across subsistence strategies or group contexts, with two exceptions – military group conflicts were biased towards between-group contexts and religious groups biased towards within-group contexts. The strongest predictors of conflict-resolution services were other prosocial functions and included group representation and providing counsel, protection and punishment, as well as qualities of interpersonal skills and fairness. Followers received social service benefits and reduced risk of harm. For leaders who resolve conflicts, status and social benefits were potential negative predictors. These results provide a comparative view of the correlates of conflict resolution suggesting diversity across social contexts.

## Graphical abstract

**Figure:**
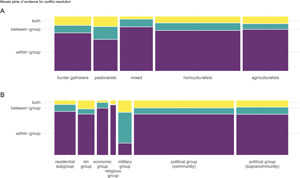

**Social media summary:** Across cultures conflict resolution is more associated with kin group than political leaders.

## Introduction

Humans are exceptional for our abilities to sustain large-scale cooperation, including between kin, co-residents, strangers and groups (Santos & West, 2018). Diverse non-human social species also demonstrate substantial cooperation (e.g. Allee, 1951). Cooperative relationships, however, are vulnerable and can decay if conflicts between individuals or groups go unresolved. Group living increases opportunity for inter-individual conflict and, across mammals, conspecific killing is most frequent among species that engage in territorial defence, and among territorial primates especially (Gómez et al., 2016). Unsurprisingly, dispute settlement mechanisms are widespread across group-living species and conflict resolution is a universal feature of human sociality (Brown, 1991; Aureli et al., 2000).

The characteristics of individuals who resolve conflicts, the associated costs and benefits of conflict resolution and the contexts that present greater conflict resolution demands have been widely discussed across disciplines, but empirical evidence from a representative sample of human social and cultural diversity has remained unavailable. Through exploratory analyses the present study aims to provide new cross-cultural insight into these empirical gaps.

### Sociality and demands of conflict resolution

Most species, including about 70% of mammals, do not live in groups (Wilson & Reeder, 2005) and the evolution of group living faces significant challenges including opportunities for conflict. Individuals in close proximity can be in conflict, for example, over access to material resources, mating opportunities and territory (Parker, 2006; E. A. Smith, 1985; Ross, 1983). Co-residents and kin can develop additional conflicts within a social structure including over position in a status hierarchy or in economic exchange (Parker et al., 2002; Hames, 2015). Humans also develop coalitionary and inter-group conflicts across levels of social organisation (e.g. kin, residential or political levels; Roscoe, 2009; Glowacki et al., 2020; Redhead & von Rueden, 2021).

Despite these challenges, for a minority of mammals including humans and most primates, group living is obligatory (Hrdy, 2007). Group living then must have in some contexts offered individuals a net fitness benefit over evolutionary history (Alexander, 1974). Putative benefits include reduced predation risks, coordination to accomplish highly profitable yet difficult goals and increased abilities to control territory (Willems & van Schaik, 2017; Smith et al., 2012). In the context of group living, co-residents who effectively solve inter-individual conflicts can benefit both at the individual level via reduced aggression (direct and indirect) and increased cooperation (Alexander, 1974; Chapman & Valenta, 2015). Groups also stand to benefit from conflict-resolution mechanisms via cultural group selection processes and mutually beneficial cooperation (Richerson et al., 2016).

### Leadership in conflict resolution

Although conflicts are common, conflict-resolution mechanisms are also common and often associated with a leadership role. Theoretical models suggest that cooperative dynamics can emerge in conflict-prone groups in the context of inter-individual heterogeneity. For example, dominant leaders, or individuals who maintain influence via aggressive or coercive strategies (Mesterton-Gibbons & Dugatkin, 1995; Cheng, 2019), may more effectively enforce norms, levy punishments and provide conflict-resolution services than non- or less dominant individuals (Mesterton-Gibbons et al., 2011; Redhead et al., 2021). Such services, however, also present opportunities for individual costs and collective action dilemmas. Despite dominance, high status or leadership status, third-party individuals who mediate conflicts may face reputational costs (Raihani & Power, 2021) and counter-punishment or aggressiveness from the individuals in conflict if the mediation is perceived as unjust (Jensen, 2010; Bøggild & Petersen, 2016). Between-group dynamics, including competition between dominant or high-ranking individuals, can also promote within-group cooperation and group investment by high-ranking individuals, which could include costly conflict-resolution services (Gavrilets & Fortunato, 2014).

Evolutionary anthropologists have emphasised the role of prestigious individuals, senior kin and other influential community members as mediators of conflicts within and between kin groups across cultures, and have suggested that conflict-resolution processes underlie much of biological and cultural evolution (Glowacki & von Rueden, 2015; Garfield et al., 2019a; Boehm, 2013). Drawing on ethnography and empirical field data from smaller-scale, politically autonomous populations, Glowacki and von Rueden (2015) frame within-group conflict resolution as one type of collective action problem groups must overcome. They suggest that (a) effective leadership emerges and is selected for owing to demands of resolving collective action conflicts within institutional systems, (b) individuals with wider social networks, greater knowledge and physical formidability will be best equipped to effectively resolve inter-individual conflicts, and (c) institutionalised leadership (i.e. culturally transmitted norms proscribing by whom and how leadership operates) probably first emerged to facilitate conflict resolution within kin groups.

Smith et al. (2016) provide a cross-species and cross-cultural comparison of leadership in within-group conflict resolution, contrasting non-human animal societies with small-scale human societies. Across their sample, within-group conflict resolution was not widely distributed, i.e. a few individuals provided conflict-resolution services among both non-human and small-scale human societies, implicating the role of individual leaders, or individuals with disproportionate group influence (von Rueden & van Vugt, 2015), in the resolution of within-group conflicts. Smith et al. (2016) also found that among both non-human and small-scale human societies leaders and followers generally equally benefited from within-group conflict resolution and conflict-resolution roles were more often achieved than ascribed, suggesting that group dynamics shape the qualities of leaders who resolve conflicts. In small-scale human societies, however, leaders who resolve within-group conflicts are more likely to use coercive power (Smith et al., 2016). Followers then are at risk of exploitation via mediation processes (as well as decision-making hierarchies, generally) and influential individuals such as community leaders may also initiate conflict in an effort to implement policies (Boehm, 1999, 2008).

Given the universality of conflict resolution across human populations and its broad phylogenetic distribution, there are likely to be evolved psychological mechanisms facilitating conflict-resolution strategies, as well as facultative responses to ecological conditions and culturally evolved systems exhibiting variability and convergence. Current perspectives highlight the need for additional research on conflict resolution and leadership across cultures and contexts. Moreover, the cultural and social diversity of within-group conflict resolution is not well documented, given that much of the evolutionary social science on conflict has focused on warfare and between-group conflict (e.g. Glowacki et al., 2020; Sluka, 1992; Lopez, 2020).

## Study aims

Conflict resolution occurs among every human society but we currently know very little about whether such processes utilise similar or variable social mechanisms (e.g. coercion or persuasion) and if they rely on the same individual characteristics across the diversity of human social and cultural contexts (e.g. high social status, large social networks, or fairness). Addressing such questions requires a broad, comparative and cross-cultural framework. Based on current theoretical and empirical literature, outstanding questions on human conflict resolution include: (a) what are the underlying behavioural, personality or other characteristics that characterise leaders who resolve conflicts across diverse cultural and social contexts; (b) what are the specific costs or benefits individuals incur from conflict-resolution processes; and (c) how widespread is coercive authority or prosociality of leaders who solve disputes, across diverse societies? Lastly, (d) does group context or cultural typology predict variation in human conflict resolution? The present study aims to provide some insight to these questions through an exploratory analysis leveraging a novel cross-cultural database (Garfield & Hagen, 2019).

Conflict resolution was the most cross-culturally frequent function of leaders, documented in 78% of cultures in a large sample of ethnography from 59 cultures (Garfield et al., 2020). Given the frequency of conflict resolution in the ethnographic record and important outstanding questions, further analyses are warranted. The current study focuses in detail on the ‘conflict resolution’ variable in the Garfield and Hagen (2019) database to identify the characteristics of leaders who resolve conflicts and the social and cultural contexts most strongly associated with conflict scenarios. The correlates of conflict resolution speak to the behavioural and phenotypic traits of individuals who resolve conflicts and to the cultural views of conflict resolution across human societies. Individual, group and culture-level measures will be used to predict evidence for conflict resolution to identify how conflict resolution may covary with cultural and social ecology. Lastly, text analysis is used to provide insight into the semantic content of the ethnographic record of conflict resolution.

## Methods

### Cross-cultural database

This study uses the *leadership data package* (Garfield & Hagen, 2019), a recently developed cross-cultural database designed to capture a wide range of ethnographic content related to leadership from the 60-cultural Probability Sample Files (Naroll, 1967) of the electronic Human Relations Area Files (eHRAF). The eHRAF is an electronic database of primary ethnographic documents which can be queried using a thorough subject code system (Outline of Cultural Materials or OCM codes) and/or by keyword, at the paragraph level. The *leadership data package* is based on 1212 ethnographic paragraphs (termed text records) extracted from the eHRAF using a broad search strategy targeting general descriptions of leadership. These text records stem from from 321 documents describing 59 cultures (see Figure S1 for the geographic distribution of the culture sample). The data package includes researcher-coded measures of evidence for 109 dimensions of leader qualities and functions (coded as ‘evidence for’ = 1 or ‘no evidence’ = 0), including conflict resolution (the *Resolve conflict* variable), as well as measures of costs and benefits for leaders and followers, a measure of group context (see Table 1), the context of leader functions (i.e. within-group, between-group or both) and culture and document-level metadata (e.g. subsistence strategy, year of publication, author). See Tables S1 and S2 in the Supplementary Information for operational definitions of all leadership dimensions.

**Table 1.** Operationalisation of the group context variable. Reproduced from Garfield et al. (2020).

Group context	Description
Residential subgroup	Informal groups of co-residents, social groups, age-based groups or performance groups
Kin group	Groups based on kin relationships, such as lineages, phratries, and clans
Economic group	Subsistence groups, market groups, and other groups with primarily economic goals
Military group	All groups related to inter-group conflict
Religious group	Groups formed for spiritual or supernatural purposes
Political group (community)	Political groups at the level of the community, i.e. political leaders, such as village headmen, can potentially interact directly with most community members
Political group (supracommunity)	Political groups that encompass multiple residential communities, such as complex chiefdoms, regional political leaders and kings or state-level leaders

As example, the following text record from Pospisil (1963) on the Kapauku agriculturalists of the Indonesian Central Highlands was coded as providing evidence for the functions of *Resolve conflict* and *Punishment*, with the group context of *kin group* and the context of conflict as *within-group*:

The behavior of members of a sublineage (or of a non-subdivided lineage) discloses mutual affection and a strong sense of belonging and unity. Within this group all fighting is considered deplorable. Even an organised stick fight, which occasionally disrupts the otherwise cordial relations within a lineage, is unheard of in this subgroup. The main responsibility of the leader of the sublineage is to prevent or to quell an occasional brawl and to mete out a deserved punishment in accord with customary law.

For additional details on database construction see Garfield et al. (2019a, 2020).

### Data analysis

The first goal of the current study was to was to identify which of the other 108 leadership dimensions were most strongly associated with evidence for conflict-resolution processes. A logistic elastic net regression model was fitted with all leadership dimensions as predictors of evidence for the *Resolve conflict* measure. Elastic net regression models are penalised regression models that are effective when the number of predictors is large relative to the number of observations. Elastic net regression models in the current study used the glmnet package (Friedman et al., 2010) and the ‘lasso’ penalty (*α* = 1), which will often set many coefficients to 0, thereby identifying the most important predictors among all covariates. Following standard procedure, 10-fold cross-validation was used to find the optimum value of the penalty term *λ*, i.e. the value that minimised cross-validation error. A second value of *λ* was also selected that was the largest value of lambda such that the error was within 1 standard error of the minimum, i.e. one that would increase shrinkage relative to the optimal *λ* and therefore decrease false positives. For the elastic net regression model of *Resolve conflict* (coded as 0 for no evidence, 1 for evidence for), coefficients from both the optimal *λ*_min_ model and the more conservative *λ*_1*SE*_ model are reported. All variables were centred and standardised by one standard deviation prior to fitting. See the Supplementary Information for further details on elastic net models.

The second goal was to assess social and cultural variation associated with evidence for conflict resolution, namely by subsistence strategy (see Figure S1) and group context (see Table 1). This goal involved two methods. First, ethnographic *evidence for* conflict resolution (i.e. *Resolve conflict*=1) was descriptively represented in relationship to group context (e.g. kin groups vs. political groups), subsistence type (e.g. hunter–gatherers vs. agriculturalits), and the type of conflict (i.e. within-group vs. between-group conflicts). Second, the *Resolve conflict* variable was used as an outcome in a logistic mixed effects regression model using the lme4 package (Bates et al., 2015) with subsistence type, group context and continental region as predictors.

The third goal was to empirically describe the ethnographic record of human conflict resolution through text analyses. Ethnography-based data are rooted in bodies of text produced by ethnographers and other authors. The semantic content of these documents can be analysed using text analytic methods to supplement analyses from researcher-coded variables. A document–term matrix was developed of all ‘informative’ words in the corpus of text records. The ‘informative’ words are the unique words produced by excluding stop words (e.g. ‘a’, ‘the’, ‘is’, ‘are’) and word ‘stemming’ which removes suffixes (e.g. ‘quarrelling’, ‘quarrelled’, ‘quarrels’ are all reduced to ‘quarrel’). In this matrix each row corresponds to one of the 1212 text records in the sample and each column to one of these 9656 unique ‘informative’ words. Each cell value in the document–term matrix indicates the number of times that word appears in that text record. Included alongside the document–term matrix is a column for the researcher-coded *Resolve conflict* variable. An elastic net logistic regression model (with the lasso penalty, *α* = 1) of *Resolve conflict* was fitted as a function of the frequencies of all 9656 words. Words that were strong positive predictors exemplify the semantic content of the text records which provided evidence for conflict resolution.

All analyses were conducted with R version 4.0.2 (2020-06-22).

## Results

The number of text records per culture in the *leadership data package* sample ranged from 1 to 126, with a median of 13, a mean of 20.5 and a standard deviation of 23.4. The text records were generally short, ranging from 14 to 1402 words, with a median word count of 141, a mean of 159.5 and standard deviation of 97.4. Publication dates of documents in the *leadership data package* ranged from 1860 to 1999, with a median year of 1964. There were 217 male authors, 62 females authors and three authors whose gender was not determined (some were co-authors). Of the 321 source documents, 70 (22%) had a female author or co-author. The total pages of the eHRAF ethnography on each culture in the culture sample ranged from 934 to 11,234, with a median of 2771, and this was weakly correlated with the number of text records on leadership retrieved for a particular culture (*r* = 0.24, *p* = 0.062). For additional descriptive results of ethnographic paragraphs in the *leadership data package* see Garfield et al. (2020), including analyses of potential bias by ethnographer gender, year of publication and total pages of ethnography per culture (overall, potential biases from these measures were deemed to be negligible).

### Leadership dimensions predictive of conflict resolution

An elastic net logistic regression model (with the lasso penalty, *α* = 1) of *Resolve conflict* was fitted as a function of the other 72 quality and function leadership dimensions, and the 36 leadership costs and benefit measures (nine measures which include cost and benefit variables for both leaders and followers, i.e. 9 × 2 × 2). This model identified which dimensions are associated with evidence for conflict resolution in this sample of the ethnographic record of leadership.

**Figure 1.**
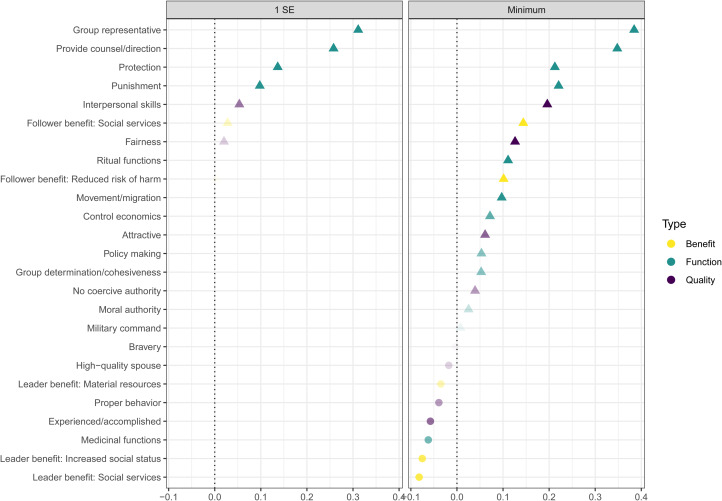
Non-zero coefficients of leadership dimensions that predicted evidence for *Resolve conflict* using the lasso penalty (*α* = 1), with *λ*_*min*_ (value = 0.01) chosen by cross-validation. Coefficients from *λ*_1*SE*_ (value = 0.03) are those under *λ* values within one standard error from *λ*_*min*_. The colour indicates leadership dimension type (for illustration only). Shape indicates positive vs. negative predictors. Point transparency is proportional to coefficient value for low values. Predictors with coefficients = 0 are not displayed. *x*-Axes are log odds on the response scale.

The non-zero coefficients under the more conservative *λ*_1SE_ indicated that the functions *Group representative* and *Provide counsel/direction* were the strongest positive predictors and the functions *Protection* and *Punishment* as well as the qualities *Interpersonal skills* and *Fairness* were moderate or weak positive predictors of evidence for *Resolve conflict* (Figure 1). The *λ*_min_ estimates also identified a suite of other qualities, functions and benefits, including *Ritual functions* and *Control economics* and the follower benefits of *Social services* and *Reduced risk of harm*. Interestingly, two benefits for leaders, *Increased social status* and *Social services*, were negative predictors under the *λ*_min_ model.

### Social and cultural variation in evidence for conflict resolution

#### Descriptive results of conflict resoultion by subsistence and group context

**Figure 2.**
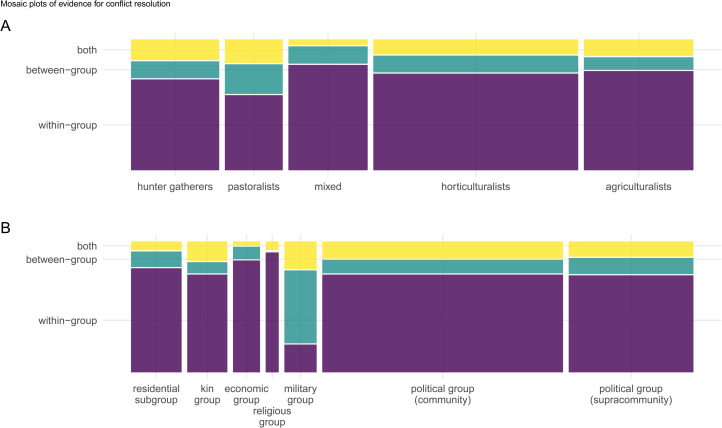
Mosaic plots of the distribution of evidence for conflict resolution. The *y*-axis and colour indicate the *context of conflict* coding. (a) By subsistence and the type of conflict. (b) By *group context* and type of conflict. Areas of the bars are proportional to the number of text records providing evidence for conflict resolution in that category.

Of the 1212 text records, 152 provided evidence for conflict resolution. The distribution of evidence for conflict resolution across subsistence type and group context (see Table 1) and in relationship to the context of conflict (e.g. within-group, between-group) is depicted in Figure 2, for these 152 text records. The *Context of conflict* was coded as ‘within-group’, ‘between-group’, ‘both’, or ‘unknown’ (‘unknown’ was only coded for three text records and has been removed from these figures).

In the mosaic plot in Figure 2 the area of each bar is proportional to the number of text records providing evidence for *Resolve conflict* in that category. The *x*-axis reveals the unbalanced evidence across subsistence types in Figure 2a and across group context in Figure 2b. This plot also reveals that across cultures with distinct subsistence types (Figure 2a), the ethnography of conflict resolution does not substantially vary across within- or between-group conflicts and there is a substantial bias in favour of evidence for within-group conflict resolution, with the possible exception that pastoralists demonstrated slightly more relative evidence for between-group conflict resolution than other subsistence groups. Across all subsistence types, however, there is evidence of between-group conflict resolution.

Across group contexts there are also general similarities in the distribution of within- and between-group conflict resolution (Figure 2b), with two obvious exceptions: leaders of religious groups primarily resolve within-group conflicts (with marginal evidence of conflicts involving both contexts) and leaders of military groups are primarily involved in between-group conflict resolution, although there is evidence of within-group conflict resolution among military leaders as well.

#### Predicting variation in conflict resolution

In their systematic analyses of all 109 leadership dimensions in the *leadership data package*, Garfield et al. (2020) identified conflict resolution as a candidate universal of human leadership (see their table 1), given that this measure did not meaningfully vary by continental region, subsistence strategy, group context or leader sex – four predictors applied to all leadership dimensions – relative to an intercept-only model. There is very little evidence for female leaders in conflict resolution in the database, however. Therefore, to expand on this modelling approach specifically for conflict resolution, a logistic mixed effects model of *Resolve conflict* was fitted (similarly to the Garfield et al. (2020) method with random intercepts for document authors nested within cultures), but which excluded the *leader sex* term. This three-term model *did* show a modest improvement in fit (using the cut off of Δ*AIC* < −2; Burnham & Anderson, 2002) over the intercept-only model (including the random intercepts), Δ*AIC* = −2.33.

**Figure 3.**
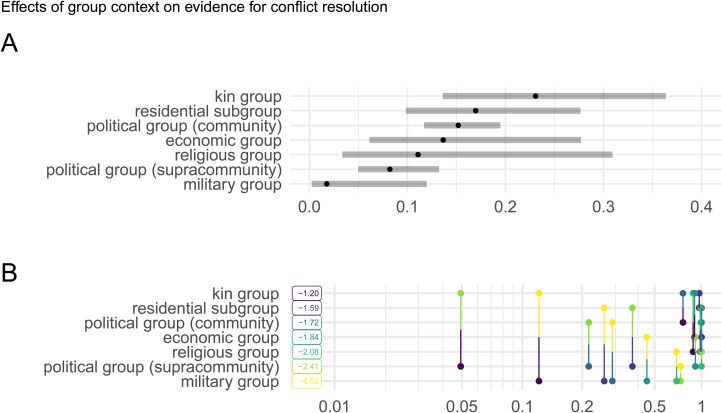
Effects of group context predicting evidence for conflict resolution (controlling for subsistence and region). (a) Estimated marginal means of evidence for conflict resolution by group context from a three-term logistic mixed-effects regression model with random intercepts for author nested within culture. Values are on the response scale (probability). (b) Contrast analysis plot from pairwise comparisons of estimated marginal means of levels of group context predictive evidence for conflict resolution (controlling for subsistence and region). Values are Tukey-adjusted *p*-values.

Figure 3a plots the estimated marginal means of *group context* from the three-term logistic mixed effects model of *Resolve conflict* and Table S2 reports results of an ANOVA test of the fitted model. *Continental region* and *subsistence type* were not significant predictors of *Resolve conflict*; however *group context* was a significant predictor (*p* = 0.02, *α* = 0.05). Pairwise comparisons of estimated marginal means from levels of the *group context* measure were compared using contrast analysis to identify the contexts which predicted evidence for *Resolve conflict* in the three-term model (Figure 3b). One pairwise comparison – *kin group* vs. *political group (supracommunity)* – produced a statistically significant mean difference (Tukey-adjusted *p*-value <0.05) with *kin groups* producing the higher estimated marginal mean.

### Text analysis of conflict-resolution ethnography

**Figure 4.**
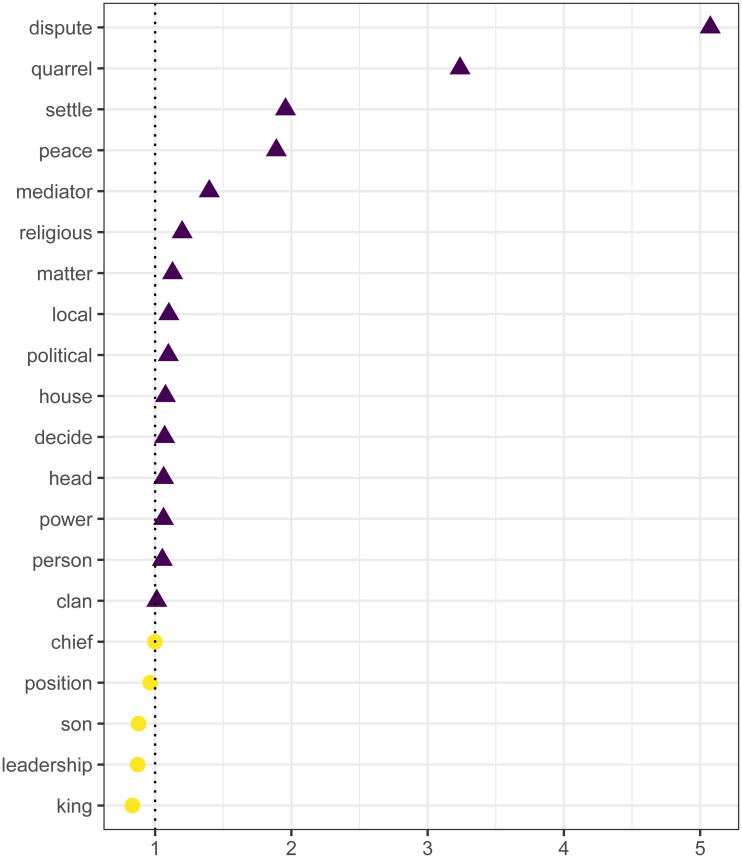
Non-zero coefficients of logistic elastic net text analysis regression model of evidence for *Resolve conflict*. Coefficients indicate the words whose frequencies in each text record best predicted evidence for *Resolve conflict* in each text record. Purple triangles indicate positive coefficients. Yellow circles indicate negative coefficients.

To empirically characterise the ethnographic record of conflict resolution, a document–term matrix was created of all ‘informative’ words in the corpus of text records and the frequency with which they occurred in each text record (see Methods). The strongest positive predictors (terms) included, unsurprisingly, ‘dispute’ and ‘quarrel’, describing conflict scenarios. The next group of positive predictors included, ‘settle’, ‘peace’ and ‘mediator’, describing resolution processes. Other notable positive predictors included, ‘decide’, implicating decision-making processes by leaders and a few terms specific to social contexts, such as ‘religious’ and ‘political’ supporting modelling results that social context is often mentioned in descriptions of conflict resolution. Several weak positive predictors were related to local and family contexts. Terms associated with more institutionalised and hierarchical leadership systems tended to be negative predictors. For example, ‘king’, ‘leadership’ and ‘chief’ were negative predictors, suggesting the ethnographic record of leadership among kingdoms and more stratified social contexts is less likely to discuss conflict resolution. See Figure 4.

## Discussion

Identifying the traits commonly associated with conflict mediators has received substantial focus and is of broad multidisciplinary interest. The vast majority of studies on the behavioural and personality traits of conflict mediators, however, have relied on Western participants in either general or very specific postindustrial contexts (e.g. Moberg, 1998; Sternberg & Soriano, 1984; Corneliussen et al., 2017). Empirical support for such traits from a representative sample of human cultural diversity has not previously been available. Furthermore, most cross-cultural and ethnographic studies of conflict and conflict resolution have focused on inter-group conflicts (e.g. Ember & Ember, 1994; Glowacki & Wrangham, 2015; Ericksen & Horton, 1992; Fry & Söderberg, 2013), leaving a gap in the cross-cultural evidence for traits associated with conflict resolution within groups. The present study aimed to provide some insight towards filling these gaps.

### The correlates of conflict resolution

Tehrani and Yamini (2020) provide a meta-analysis of psychological studies investigating associations between conflict resolution and personality traits (using the Five-Factor Personality Inventory) with five distinct conflict-resolution styles: avoidance, compromising, integrating, obliging and dominating. Relevant for the pattens observed here, they found (a) a positive relationship between extroversion and the dominating style of conflict resolution – where conflict mediators display a high concern for self, a low concern for others, and are selfish in influencing outcomes – and (b) agreeableness negatively associated with dominating styles and positively associated with both an obliging style – where conflict mediators display a low concern for self and a high concern for others, and promote prosocial outcomes – and an integrating style – where conflict mediators display a high concern for self and others and promote mutually beneficial outcomes.

Results here on the importance of providing counsel and direction, interpersonal skills and fairness among conflict mediators across cultures and contexts (Figure 1a) support the relationships between agreeableness and the integrating and obliging styles of conflict resolution identified by Tehrani and Yamini (2020). Other group-service provision functions are more strongly related to evidence for conflict resolution in the ethnographic record than are many other theoretically important qualities. The association of providing counsel and direction in followers affairs, rather than evidence for coercion, fear, manipulation or physical formidability, suggests that conflict resolution often involves leaders advising followers and providing input *influencing* outcomes rather than *mandating* specific outcomes. The ethnographic evidence suggests that, across cultures, conflict resolution is less likely to be associated with coercive dominance-based influence and more likely to be associated with freely conferred prestige-based influence (Redhead et al., 2021). Leaders who resolve conflicts are also likely to perform other prosocial group functions including group representation, counselling and advising followers, and providing protection and punishment services. Counselling followers in conflict is also key *computational service* and the connection between counselling and conflict-resolution services lends some support to the importance of mutually beneficial decision-making cognition as a potential selective pressure in evolutionary models (Garfield et al., 2019a; Hagen & Garfield, 2019).

Analyses here did not find an association between dominance-based leadership and conflict resolution. However, Garfield et al. (2020) (using the same data analysed here) found that providing punishment services was the strongest positive predictor of leaders with coercive authority, a quality strongly implicated in the dominance style of leadership (Cheng, 2019) and punishment services by leaders was moderately associated with conflict resolution in results here. Although qualities linked to dominance-based influence may not be strongly or directly associated with resolving conflicts in the ethnographic record, there is potentially an indirect connection between abilities to enforce punishment and resolve conflict, with dominance-based influence (Redhead et al., 2021).

Empirical and ethnographic findings across diverse societies, including relatively egalitarian populations, suggest that group representation often includes the development and maintenance of cooperative relationships and alliances (Boehm, 1999; von Rueden et al., 2014; Bowser & Patton, 2010). The importance of group representation in conflict resolution generally lends some support to Boehm's (1982: 329) speculation that, ‘when peace is actively created between fighting groups, it is likely that the same ideologies that so strongly support conflict resolution within the group are being applied in a similarly problem-solving fashion, but obviously in a between-group context’.

It is worth noting that the dimensions in the *leadership data package* capturing social capital, knowledge, experience or decision-making capacities, or physical formidability were not identified as predictors of conflict resolution, as might be expected given related empirical and theoretical work (e.g. Glowacki & von Rueden, 2015; Garfield et al., 2019a; Hagen & Garfield, 2019; although see ‘Limitations’). Interpersonal skills and fairness were the only leader qualities predictive of conflict resolution. This supports links between effective conflict resolution and moral evaluations of social justice, suggesting that individuals who conform to and embody such traits are preferred as mediators. Effective mediators of conflict then are not necessarily leaders who can be conceptualised as particularly prestigious or dominant but are more likely to be individuals who effectively identify overlapping interests between individuals with distinct priorities fairly, consistent with emerging views on leadership and followership focused on the process and outcomes over individual traits (Vollan et al., 2020; Wiessner, 2019). Strong preferences for fairness potentially have deep evolutionary origins (Stavans & Baillargeon, 2019; Nowak et al., 2000; Haidt, 2007). The importance of these leader qualities supports an evolved psychology of procedural fairness and evaluations of welfare trade-off ratios, or psychological preferences for leaders who will appropriately weight individual welfare in conflict resolution decision-making processes (Petersen et al., 2010; Sell et al., 2009; Bøggild & Petersen, 2016).

### Benefits associated with conflict-resolution processes

There were two benefits for followers associated with conflict resolution in the ethnographic record: *Social services* and *Reduced risk of harm*. These variables effectively identify the conflict-resolution process itself. Two leader benefits, *Increased social status* and *Social services*, were negative predictors of evidence for conflict resolution. This suggests that the well-known ethnographic descriptions of high-status community leaders and the special services and privileges that they receive are not often linked to descriptions of conflict resolution. Conflict resolution then might best be viewed as a prosocial service that leaders or other individuals provide, for which they do not receive substantial direct benefits. If so, then this view fails to support models of reciprocal exchange of social service (e.g. conflict resolution) for social benefits or mutually beneficial outcomes (Price & Van Vugt, 2014; Hagen & Garfield, 2019; Garfield et al., 2019a). Alternatively, given the importance of conflict resolution in kin groups (see below), much of the ethnographic record of conflict resolution might be better understood as an investment in inclusive fitness or kin group welfare rather than a strategy for increased social mobility or personal gain (Hames, 2015).

### The cultural ecology of conflict resolution

Anthropologists have long debated the relative importance of concerted conflict management across populations with varying levels of ‘complexity’, generally defined as more intensified subsistence economies and more elaborated and hierarchical sociopolitical structures (cf. Knauft et al., 1991; Wiessner, 2016; Lee, 1972). For example, Lee (1972: 182), downplaying within-group conflicts and conflict resolution among mobile hunter–gatherers suggests that:

in contrast to agricultural and urban peoples, hunters have a great deal of latitude to vote with their feet, to walk out of an unpleasant situation. And they do so, not when the food supply is exhausted, but well before that point when only their patience is exhausted. This mobility has a profound ecological adaptive significance. Fear and avoidance of conflict has the effect of keeping people apart.

Demography is also implicated in shaping conflict rates and management and Lee (2018: 6) suggests that ‘living at very low densities, foragers had fewer things to fight over and, with little or no fixed property, could easily vote with their feet and disperse to diffuse conflict’. Larger more densely populated, sedentary groups with more intensified economies and greater sociopolitical complexity are thought to have greater pressures of within- and between-group conflicts, and hence be equipped socially and politically with more elaborate conflict-management processes. Despite any influence on conflicts owing to mobility and demography, conflict-resolution and -management systems are universal across human societies, including among mobile hunter–gatherers (see Boehm, 2013). The ‘vote with their feet’ hypothesis would suggest evidence for conflict resolution to be more frequent in the ethnographic record of agricultural and horticultural populations relative to more mobile hunter–gatherers or pastoralists. Exploratory analyses here, however, did not find variation in evidence for within- or between-group conflict resolution across cultures with different subsistence strategies (Figure 2a) and subsistence type did not predict evidence for conflict resolution (controlling for region and group context, Figure 3). Therefore, the ‘vote with their feet’ hypothesis promoted by Lee and others and a cultural ecology of conflict resolution grounded in variation in subsistence strategies are not supported. One possible exception to this trend was the slightly greater frequency of evidence for between-group conflict resolution among pastoralists (Figure 2a). This could be due in part to pressures from shared resources related to livestock-based subsistence economies including access to water sources and grazing land and the diffuse and shifting nature of territoriality among pastoralists (Glowacki & Gonc, 2013; Garfield et al., 2019b; Fratkin, 2014).

Controlling for subsistence strategy and continental region, group context was a significant predictor of evidence for conflict resolution, with kin groups providing greater evidence for conflict resolution in contrast to higher-order political groups (Figure 3). Logistic regression identified a general trend of greater evidence for conflict resolution among more local and generalised groups (e.g. kin group, residential subgroups) compared with larger and more specialised groups (e.g. hierarchical political groups, military groups), which is supported by the text analysis of conflict-resolution ethnography (Figure 4). This trend suggests multiple, non-mutually exclusive interpretations. Leaders of higher-order political groups may have other more demanding tasks than intervening in conflicts between group members and kin groups may experience greater demands or have a greater interest in resolving conflicts. Another set of interpretations would suggest that a stronger influence of social institutions among populations with more developed political structures functions to resolve conflicts with limited involvement of third-party individuals. Also, among more despotic and socially stratified societies, many potential conflicts may not manifest behaviourally given extreme power asymmetry between aggressor and victim. Future research should work to disentangle causal relationships between rates of conflicts by group context, social and political structures, and leadership functions.

Lastly, the ethnographic record of conflict resolution provides greater evidence for conflict resolution within groups than between groups, including across populations with variable subsistence strategies and across distinct social contexts (Figure 2 and see ‘Limitations’ section). These results are consistent with recent work in anthropology emphasising heterogeneity, variability and social complexity among populations such as mobile hunter–gatherers, often characterised as ‘small-scale’ (e.g. Hill et al., 2011; Bird et al., 2019; Singh & Glowacki, 2021). Between-group conflicts, although relatively less represented, are consistently documented across all subsistence types including hunter–gatherers, providing additional evidence for the ubiquity of inter-group conflict across the vast majority of human societies (Glowacki et al., 2020; Hames, 2019; Lopez, 2016).

### Limitations

The current study has many important limitations. First, ethnography-based analyses are necessarily restricted to the content that ethnographers chose to record and publish. It is important to keep in mind that information that ethnographers were unaware of, disinterested in or not permitted to research constrains available information in any ethnographic document. For example, the biased evidence in favour of within-group conflicts could entirely be an artefact of ethnographer description. Furthermore, much (or most) ethnography is constructed from the perspective of the ethnographer (an emic view) and not necessarily from the perspective of members of the focal population (an etic view). Ethnographers’ perspectives on conflicts could be divergent from those of local community members.

Given the general content of text records in the *leadership data package* these results are biased towards particular types of leaders, generally male political leaders. As previously mentioned these data include very few text records pertaining to women as mediators of conflict. Results from this comparative, ethnography-based dataset are also unable to provide direct comparison with related empirical and field-based research owing to ecological framing differences, measurement and sampling differences, and the aforementioned limitations.

All analyses conducted here are exploratory and correlational and do not suggest that particular leadership dimensions cause conflict-resolution capacities or vice versa. Also, these data cannot disentangle the degree to which leaders may be actively involved in, have a vested interest in, or cause the conflicts in which they have been described in association with. Given these limitations, these results can provide a comparative, empirical foundation for future more detailed studies on the context of conflicts and their interactions with traits associated with conflict mediators.

## Conclusion

The ethnographic record provides evidence that conflict resolution by leaders often occurs in the context of within-group conflicts across populations with variable subsistence economies. Evidence for between-group conflict resolution, although more limited, is also not biased towards any particular subsistence strategy. Culturally and economically diverse populations probably face similar demands for conflict resolution and individual leaders reliably emerge as conflict mediators across human societies.

Conflict resolution services are perhaps best viewed as part of larger leadership processes aiming to promote within-group cohesion and strategic, beneficial between-group relations. Variation in conflict-resolution capacities are more likely to be associated with a wider range of leadership functions, such as representing the group, providing counsel, protecting group members and punishing defectors, than particular personality traits or individual qualities. The qualities of interpersonal skills and fairness, however, are potentially important for effective conflict resolution across diverse cultural and social contexts. These results suggest that followers benefit primarily directly from conflict-resolution processes, and there was no direct evidence that mediators of conflict receive reciprocal benefits such as special status or reciprocal services from followers. Although evidence for conflict resolution did not meaningfully vary across regions or by subsistence strategies, there was some evidence that group context impacts demands for inter-individual conflict resolution. More localised and smaller groups, such as kin groups more often feature leaders who actively resolve conflicts. Leaders within higher-order political groups are less likely to be actively involved in conflict resolution.

The ethnographic record is an indispensable tool for uncovering cultural diversity and universality. To better understand the potential for conflict emergence, scholars and managers should focus on micro-level group and social contexts, rather than macro-level group variation, such as subsistence economies or national characteristics. Groups with less hierarchical structure and greater face-to-face interaction may be predisposed to increased rates of within-group interpersonal conflicts. Individuals who generally provide group-beneficial services and have reputations for fairness and interpersonal skills may be best equipped to facilitate conflict resolution. Alternatively, increased internal organisational structure may also reduce the likelihood of individual conflicts emerging and facilitate conflict mediation structurally. Theoretical models of conflict resolution can draw on the systematic results here in developing more robust, generalisable theories of conflict management.

## Supplementary material

For supplementary material accompanying this paper visit https://doi.org/10.1017/ehs.2021.41. click here to view supplementary material

## Research transparency and reproducibility

The data associated with this project are available at https://doi.org/10.5281/zenodo.2541999.
