# Supplementary material for: Correlates of conflict resolution across cultures
Source: Evol Hum Sci. 2021 Aug 31;3:e45. doi: 10.1017/ehs.2021.41 (PMC10427275; doi:10.1017/ehs.2021.41)
Supplement: Supplementary file 1 [file S2513843X21000414sup001.docx]

Correlates of conflict resolution across cultures

Zachary H. Garfield

^1^ Institute for Advanced Study in Toulouse, Université de Toulouse 1 Capitole, Toulouse, France

[zachary.garfield@iast.fr](mailto:zachary.garfield@iast.fr)

## Supplementary information

### Culture sample

Figure S1: The geographic distribution of the culture sample. Symbol shape indicates subsistence strategy and symbol size indicates the number of documents available for that culture. Axes are degrees latitude and longitude. Reproduced from Garfield et al. (2020).

## Operationalization of Leader Qualities and Functions variables

Table S1: Operational definitions for coded leader qualities and functions.

| Type | Name | Definition |
| --- | --- | --- |
| Functions | Bestow mates | The leader organizes arranged marriages or distributes access to mates to followers. |
|  | Construction/infrastructure | The leader makes decisions or is in charge of local construction/infrastructure and maintenance of infrastructure. |
|  | Control calendar | The leader determines the calendrical system. |
|  | Control economics | The leader controls economic exchange or economic systems/production within the group. |
|  | Control immigration | The leader decides who can move into the group. |
|  | Council member | The leader is a member of a council that serves community interests or makes community decisions. |
|  | Distribute resources | The leader is in charge of distributing resources (food and non-food) to followers (independent of production). |
|  | Group determination/cohesiveness | The leader is responsible for maintaining (explicitly or implicitly) group identity; followers form distinct groups based on their affiliation with a specific leader; the leader functions to maintain group cohesiveness. |
|  | Group representative | The leader represents the group to an outgroup (or possibly to sub-groups in larger societies); can include travel to visit and maintain ties with other communities. |
|  | Hospitality | The leader hosts guests and entertains individuals in specific contexts. |
|  | Medicinal functions | The leader provides medicinal or healing services to heal physical or mental illness or injury. |
|  | Military command | The leader participates and leads in military activities, directly or indirectly. |
|  | Moral authority | The leader instructs on or serves as a model to followers for proper behavior; the leader sets standards of appropriate behavior. |
|  | Movement/migration | The leader decides when and where to move to a new settlement; controls/influences group movement. |
|  | Organize cooperation | The leader organizes collective activities and controls labor in the group (i.e., takes an administrative/managerial role in cooperation). |
|  | Policy making | The leader makes rules and decisions that group members must follow, making executive decisions; includes creating and shaping social norms. |
|  | Political appointments | The leader assigns or appoints others to sociopolitical positions. |
|  | Prosocial investment | The leader’s behavior benefits group members; the leader’s actions produce positive outcomes for the group. |
|  | Protection | The leader provides protection to followers, directly or indirectly, including from physical threats, spiritual threats, within-group protection from aggression, between-group protection from invaders/attackers (non-subsistence related). |
|  | Provide counsel/direction | The leader advises, councils others, or provides input influencing outcomes for followers (not including conflict resolution). |
|  | Provide subsistence | The leader directly produces subsistence surpluses for followers, such as through hunting, growing food, collecting food for collective consumption (independent of distribution). |
|  | Punishment | The leader sanctions or punishes transgressors of social rules/norms or free-riders. |
|  | Resolve conflict | The leader resolves disputes between followers or is sought out to resolve conflict. |
|  | Ritual functions | The leader performs ritual or religious functions or services for the group. |
|  | Serve a leader | The leader serves a higher ranked leader in some capacity or executes orders from a superior. |
|  | Social functions | The leader provides a social function (non-punishment), such as facilitating marriages, brokering economic exchange, organizing social activities, or managing group-level social events/problems. |
|  | Strategic planning | The leader makes decisions/plans for the future relying on their knowledge and intellect in order to optimize outcomes for the group. |
| Qualities | Age | The leader is an elder or is older than most followers. |
|  | Aggressiveness | The leader demonstrates use of physical or verbal aggression or has a high propensity/reputation for aggressiveness. |
|  | Ambitious | The leader is described as ambitious, determined, enthusiastic, or highly motivated. |
|  | Artistic performance | The leader is an artistic performer for followers, e.g., song, dance, other artistic performances. |
|  | Attractive | The leader is described/perceived as being physically/sexually attractive. |
|  | Bravery | The leader is known for being courageous, brave, or fearless. |
|  | Charisma | The leader is described as charismatic, able to motivate followers with their personality. |
|  | Coercive authority | The leader has strong authority over followers and social norms either promote or accept this authority; or followers are unable to resist or depose coercion of the leader. |
|  | Confidence | The leader is confident, assured, or assertive. |
|  | Culturally conservative | The leader values tradition over new/outside ideas. |
|  | Culturally progressive | The leader is open to new/outside ideas and might value these ideas over tradition. |
|  | Decisiveness/decision-making | The leader makes decisions swiftly or has a reputation for good decision-making abilities. |
|  | Dishonest | The leader has a reputation for or has demonstrated dishonesty. |
|  | Drug consumption | The leader is known for or is expected to be able to consume high levels of drugs (alcohol, tobacco, etc.), is expected to have a high tolerance, or maintain superior cognitive functioning under the influence relative to followers. |
|  | Experienced/accomplished | The leader has increased/specialized expertise relative to followers or has a reputation for successful and notable accomplishments. |
|  | Fairness | The leader demonstrates fairness to all group members or has a reputation for fairness. |
|  | Favorable personality | The leader is described as having an ideal personality for the required context. |
|  | Feared | The leader is feared by others or has a reputation for being feared. |
|  | Generosity | The leader displays or has a reputation for generosity. |
|  | High status | The leader is described as having high social status, prestige, or held in high esteem. |
|  | High-quality spouse | The leader is married to a high status or high-quality spouse(s). |
|  | Honesty | The leader has a reputation for or has demonstrated honesty. |
|  | Humility | The leader is modest in estimating or signaling their own abilities or qualities; has a reputation for being humble. |
|  | Industriousness | The leader is hardworking and productive. |
|  | Ingroup member | The leader is a member of the local group in residence and ethnicity with such membership explicitly mentioned as preferable or important. |
|  | Innovative | The leader is described as being innovative or developing novel solutions/proposals. |
|  | Interpersonal skills | The leader works well with people and can effectively manages social relationships. |
|  | Killer | The leader has killed someone. |
|  | Knowledgeable/intelligent | The leader is known for superior intelligence, knowledge, cognitive functioning, specialized education, wisdom, or esoteric knowledge. |
|  | Loyalty | The leader demonstrates loyalty to the in-group or important members of the in-group. |
|  | Many children | The leader has many offspring. |
|  | No coercive authority | The leader does not have strong authority over followers and social norms limit or prohibit coercive authority; or followers are able to resist or depose the coercion by the leader. |
|  | No drug consumption | The leader is expected to or does not consume high levels of drugs (alcohol, tobacco, etc.), or is known for abstaining from recreational drug use. |
|  | Oratory skill | The leader is a good public speaker, eloquent, demonstrates high oratory skill, or forceful/persuasive speaking abilities. |
|  | Physical health | The leader is physically healthy. |
|  | Physically formidable | The leader is physically strong, large in size, or physically formidable. |
|  | Polygynous | The leader is polygynous or has multiple current mates/sexual partners or is a member of a polygynous marriage. |
|  | Proper behavior | The leader demonstrates proper behavior for specific contexts or that is particularly valued. |
|  | Prosocial | The leader is concerned with group welfare or has a reputation for group-wide altruism and selflessness. |
|  | Social contacts | The leader has a high number of social contacts or allies. |
|  | Strategic nepotism | The leader or the leadership system strategically provides benefits to kin/family members. Social influence/leadership is partially determined or mediated via kinship or inheritance. |
|  | Strategic planner | The leader makes decisions/plans for the future relying on their knowledge and intellect in order to improve outcomes for the group; considers outcomes strategically. |
|  | Supernatural | The leader is perceived to possess supernatural qualities/abilities. |
|  | Unfair | The leader is unfair to some group members or has a reputation for not being fair. |
|  | Wealthy | The leader is wealthier than followers including cash, material, or other economic wealth measures. |
|  | Xenophobia | The leader is described as fearing and mistrusting outsiders. |

### Additional results

*Table S2: Analysis of deviance results (Type II Wald chisquare tests) of three-term model of Resolve Conflict.*

| Covariate | Chisq | Df | Pr(>Chisq) |
| --- | --- | --- | --- |
| Group context | 14.652 | 6 | 0.023 |
| Subsistence type | 7.931 | 4 | 0.094 |
| Continental region | 3.690 | 5 | 0.595 |

#### Elastic net model details and diagnostics

Unlike standard regression models, which are fit by minimizing an objective function (i.e., in logistic regression the negative log-likelihood, $-loglik(\beta)$) penalized regression models minimize the objective function *plus* a penalty term, $\lambda$ (Le Cessie and Van Houwelingen 1992). There are two popular forms of penalized regression: ridge regression and lasso regression. For ridge regression the penalty is $||\beta||_{2}^{2}=\sum_{j=1}^{p} \beta_{j}^{2}$ and for lasso regression the penalty is $||\beta||_{1}=\sum_{j=1}^{p} |\beta_{j}|$, where $\beta_{j}$ represents the regression coefficients. Therefore, when $\lambda=0$ standard estimation is applied. As $\lambda\to\infty$, the coefficients $\beta_{j}$ are “shrunk” to 0. Thus, at lower values of $\lambda$ the $\beta s$ are relatively unrestricted, which can result in good fit to the current sample (low bias), but poor fit on future samples (high variance); the model will tend to be over-fitted. At higher values of $\lambda$ the $\beta s$ tend to shrink toward 0, which reduces fit on the current sample (high bias), but results in a more stable fit across samples (low variance); the model will tend to be under-fitted. The optimal value of $\lambda$ is typically found by minimizing cross-validation error. Elastic net regression can combine the advantages of ridge and lasso penalties using an additional tuning parameter $\alpha$, $0\leq\alpha\leq1$:

$$penalty=(1-\alpha)/2||\beta||_{2}^{2}+\alpha||\beta||_{1}.$$

Thus, $\alpha=0$ applies the ridge penalty and $\alpha=1$ applies the lasso penalty. All elastic models used in the current study apply the lasso penalty. Given the large number of predictors ($n=108$) and much sparsity in the matrix for many predictors, lasso regression and greater variable selection (i.e., fewer predictors with non-zero coefficients) was determined to provide more effective interpretations. Cross-validated $\alpha$ models were explored but did not provide any additional benefit in interpretations and were much more difficult to plot and read, given many more variables with non-zero but not meaningful coefficients. No weighting was applied in elastic net models here.

The cross-validation curve of the elastic net model of conflict resolution (seen in Figure 1) is provided below (Figure S2). This plot illustrates the cross-validation associated with both $\lambda$ values and the corresponding number of non-zero coefficients under each.

Figure S2: Cross validation plot of elastic net model of Resolve conflict presented in main text.

*Table S3: Cross validation results of lambda values from elastic net model of conflict resolution.*

| **Lambda type** | **Lambda value** | **Index** | **Measure** | **SE** | **Non-zero** |
| --- | --- | --- | --- | --- | --- |
| min | 0.01080 | 24 | 0.6441 | 0.03044 | 25 |
| 1 SE | 0.03298 | 12 | 0.6738 | 0.03233 | 8 |

### Text analysis and DTM details

The text analysis aimed to identify the unique words most strongly associated with evidence for conflict resolution at the text-record level. Descriptive statistics on paragraph length are given in the main text. Intuitively, paragraph length may be confounded with terms associated with evidence for conflict resolution (i.e., longer paragraphs may be more likely to provide evidence for conflict resolution and therefore be more likely to include terms predictive of conflict resolution).

Across the document-term matrix total word count does not correlate with evidence for *Resolve conflict* (r = -0.028). Comparing correlations between total word count and the frequency of any signal word, correlations are modest: maximum correlation = 0.32, minimum correlation = -0.05, mean correlation = 0.03 with SD of 0.05. That is, there are relatively few words in which their frequency correlates with total paragraph word count (about 1.3% of the 9,656 unique words have a correlation coefficient of 0.20 or higher with total paragraph word count). The elastic net model used in text analysis (Figure 4) was not weighted and cross-validation of $\lambda$ provides the variable (e.g., word) selection.

## References

Le Cessie, Saskia, and Johannes C Van Houwelingen. 1992. “Ridge Estimators in Logistic Regression.” *Applied Statistics*, 191–201.
